# Preliminary characterization and natural history of hantaviruses in rodents in northern Greece.

**DOI:** 10.3201/eid0606.000618

**Published:** 2000

**Authors:** A Papa, J N Mills, S Kouidou, B Ma, E Papadimitriou, A Antoniadis


### Preliminary Characterization and Natural History of Hantaviruses in Rodents in Northern Greece

**To the Editor:** Hantaviruses (family Bunyaviridae), cause hemorrhagic fever with renal syndrome (HFRS) in Europe and Asia and hantavirus pulmonary syndrome in the Americas. In Greece, hantaviruses are endemic, and small outbreaks or sporadic cases of HFRS have been observed; Dobrava virus is the predominant hantavirus and causes severe HFRS (1,2), while Puumala virus is probably the causative agent of the milder forms of this syndrome (Papa et al., unpub. data). Hantaan virus, associated with *Apodemus agrarius*, and Seoul virus, associated with *Rattus norvegicus*, have never been detected in Greece. Dobrava virus is hosted by *A. flavicollis* in the Balkans (3,4) or by *A. agrarius* in northern Europe (5).

We surveyed two sites where Dobrava virus-caused HFRS was reported (2). The first was Nevrokopi, a small town in the Rhodope Mountains, 12 km from the Greek-Bulgarian border (41° 20.184' N, 23° 49.479' E, elevation approximately 650 m). The second site included Pramanta, a small village in the Pindos Mountains, approximately 67 km southeast of Ioannina (39° 31.722' N, 21° 5.872' E, elevation 790 m), and Matsuki, a small village near Pramanta (39° 33.909' N, 21° 9.713' E, elevation 1080 m). Animals were trapped and sampled outdoors according to established safety guidelines (6). Blood, lung, kidney, and spleen samples were kept in liquid nitrogen until transferred to -70°C freezers for storage. At Nevrokopi, 57 small mammals were captured during 887 trap nights for an overall trap success rate of 6.4%. At Pramanta and Matsuki, 13 small mammals were captured during 400 trap nights (3.3% trap success). The total of 70 captured mammals comprised seven species of rodents and one insectivore. *A. flavicollis* was the most commonly captured species (87% of captures).

Whole-blood specimens were tested for hantavirus immunoglobulin G by indirect immunofluorescence assay and enzyme-linked immunosorbent assay, using Hantaan 76-118 as antigen. Total RNA was extracted from homogenized tissues, and nested reverse transcriptase-polymerase chain reaction (PCR) was performed with two sets of nested primers (2): one set designed to detect the partial G1 coding region of hantaviruses associated with rodents of the subfamily Murinae (Hantaan, Dobrava, and Seoul viruses), and another to detect the N coding region of hantaviruses associated with rodents of the subfamily Arvicolinae (Prospect Hill virus). Eight *A. flavicollis*, all from Nevrokopi, were positive for hantavirus infection by serology or molecular methods, for a 13% overall prevalence in *A. flavicollis*. Some rodents positive by serology were negative by PCR and vice versa. One of three *R. rattus* captured at Pramanta was positive for hantavirus infection by indirect immunofluorescence assay.

PCR yielded products from tissues from seven *A. flavicollis*. A 270-bp segment of the G1 gene was sequenced and analyzed phylogenetically. A mean sequence similarity of 99.1% (range 98.5%-100%) was observed among the seven rodents. These sequences differed by 9.5% from Dobrava virus sequences of HFRS cases from northwestern Greece and by 8.5% from Dobrava virus sequences of HFRS cases from Dobrava-Slovenia. Similarly, sequences from Nevrokopi human samples were closer to Dobrava virus from Slovenia than to such virus from northwestern Greece. The nucleotide difference was 21% when the rodent sequences were compared to an Estonian sequence from *A. agrarius*. The deduced amino acid sequences of all seven Dobrava virus G1 fragments were identical. This analysis showed that the evolutionary relationship among Dobrava virus subtypes was closely correlated with that of the rodent reservoir and suggests that this virus is stably maintained in the rodent population.

All seropositive *A. flavicollis* were sexually mature adults; six (75%) of eight were male, compared to 26 (49%) of 53 seronegative animals ($\chi^2$=0.97, p=0.32). Six seropositive animals of 8 (75%) had scars, compared to 14 (26%) of 53 seronegative animals ($\chi^2$ =5.4, p=0.02). Finally, 14 (44%) of 32 male *A. flavicollis* had scars, compared to 5 (17%) of 29 females ($\chi^2$ =3.8, p=0.05). Scarring has been significantly associated with Seoul virus antibody in wild rats and has been suggested as the primary mechanism by which hantaviruses are amplified epizootically (7). The higher prevalence of scars among male *A. flavicollis* and especially among males with evidence of hantavirus infection supports the hypothesis that hantaviruses are transmitted when aggressive male animals fight. Although this pattern has been observed for several host species of New World hantaviruses, this is the first known demonstration for Dobrava virus and *A. flavicollis*. Of the two female rodents with evidence of hantavirus infection, one had scars, and one did not. The latter was positive by PCR on lung tissue but did not have detectable antibody in blood, which perhaps indicates very recent infection.

The seropositive *R. rattus* from Pramanta is the first evidence of hantavirus infection in *Rattus* within Greece. No *Rattus* captured during previous expeditions had hantavirus antibody (8). The low antibody titer (1:32) and failure to amplify viral RNA by PCR from this animal could indicate infection with a heterologous hantavirus with low cross-reactivity. Perhaps more likely, the antibody detected in this 39-g juvenile rat may represent waning maternal antibody. Transfer of protective maternal antibody to *R. norvegicus* pups by Seoul virus-infected dams has been demonstrated (9).

Our data implicate *A. flavicollis* as the reservoir of Dobrava virus in northern Greece and demonstrate the common occurrence of that species in both sylvatic and peridomestic habitats. These preliminary results underscore the need for continued, more intensive reservoir studies in Greece.

### Acknowledgments

We gratefully acknowledge Jon Dunnum for helping identify small mammal specimens and Stuart Nichol for helping in the phylogenetic analysis.

This study was supported by the World Health Organization, grant no. A18/286/2GRE.

**Anna Papa,* James N. Mills,† Sophie Kouidou,* Benjiang Ma,* Evagelia Papadimitriou, and Antonis Antoniadis***

*Aristotelian University of Thessaloniki, Thessaloniki, Greece; and †Centers for Disease Control and Prevention, Atlanta, Georgia, USA

### References

1. Antoniadis A, Stylianakis A, Papa A, Alexiou-Daniel S, Lambropoulos A, Nichol ST, et al. Direct genetic detection of Dobrava virus in Greek and Albanian haemorragic fever with renal syndrome (HFRS) patients. J Infect Dis 1996;174:407-10.
2. Papa A, Johnson AM, Stockton PC, Bowen MD, Spiropoulou CF, Ksiazek TG, et al. Retrospective genetic study of the distribution of hantaviruses in Greece. J Med Virol 1998;55:321-7.
3. Avsic-Zupanc T. Hantaviruses and hemorrhagic fever with renal syndrome in the Balkans. In: Saluzzo JF, Dodet B. Factors in the emergence and control of rodent-borne viral diseases. Amsterdam: Elsevier;1999: p. 93-8.
4. Papa A, Spiropoulou C, Nichol S, Antoniadis A. Tracing Dobrava hantavirus infection. J Infect Dis 2000;181:2116-7.
5. Nemirov K, Vapalahti O, Lundkvist Å, Vasilenko V, Golovljova I, Plyusnina A, et al. Isolation and characterisation of Dobrava hantavirus carried by the striped field mouse (*Apodemus agrarius*) in Estonia. J Gen Virol 1999;80:371-9.
6. Mills JN, Childs JE, Ksiazek TG, Peters CJ, Velleca WM. Methods for trapping and sampling small mammals for virologic testing. Atlanta: U.S. Department of Health and Human Services;1995.
7. Glass GE, Childs JE, Korch GW, LeDuc JW. Association of intraspecific wounding with hantaviral infection in wild rats (*Rattus norvegicus*). Epidemiol Infect 1988;101:459-72.
8. LeDuc JW, Antoniades A, Siamopoulos K. Epidemiological investigations following an outbreak of hemorrhagic fever with renal syndrome in Greece. Am J Trop Med Hyg 1986;35:654-9.
9. Dohmae K, Nishimune Y. Protection against hantavirus infection by dam's immunity transferred vertically to neonates. Arch Virol 1995;140:165-72.

### Imported Dengue in Buenos Aires, Argentina

**To the Editor:** After more than 70 years without reports of cases, an outbreak of dengue (type 2) occurred in the northwestern region of Argentina from January to May 1998; 818 cases of denguelike illness were reported (incidence rate: 45/10,000 inhabitants) (1). The outbreak was restricted to a few cities of the Chaco Salteño Region.

The last dengue epidemic in Argentina (in 1926) (2) affected the Mesopotamia Region and Rosario City. An earlier widely distributed epidemic in 1916 occurred in the coastal region along the Uruguay River (Corrientes and Entre Ríos provinces), reached Parana City (along the Parana River), and affected approximately 50% of the city's population (3). Both outbreaks began in Paraguay. No cases were detected in Buenos Aires.

High numbers of *Aedes aegypti* are reported in all places where surveillance for these vectors is conducted in Argentina. The Breteau rate (a measure of vector density; the number of positive containers is divided by the number of inspected houses) in the Federal District

